# Mongolian Medicine RuXian-I Treatment of Estrogen-Induced Mammary Gland Hyperplasia in Rats Related to TCTP Regulating Apoptosis

**DOI:** 10.1155/2019/1907263

**Published:** 2019-03-19

**Authors:** Jun-Fei Zhang, Jia Liu, Guo-Hua Gong, Bin Zhang, Cheng-Xi Wei

**Affiliations:** ^1^Medicinal Chemistry and Pharmacology Institute, Inner Mongolia University for Nationalities, Tongliao, Inner Mongolia, China; ^2^Inner Mongolia Key Laboratory of Mongolian Medicine Pharmacology for Cardio-Cerebral Vascular System, Tongliao, Inner Mongolia, China; ^3^Affiliated Hospital of Inner Mongolia University for Nationalities, Institute of Mongolia and Western Medicinal Treatment, Tongliao, Inner Mongolia, China

## Abstract

Mongolian medicine RuXian-I is composed of 30 Mongolian herbs, which is a traditional Mongolian recipe for clinical treatment of breast “Qi Su Bu Ri Le Du Sen” disease (hyperplasia of mammary glands, HMG). Based on the previous study, this dissertation further explores the therapeutic mechanism of RuXian-I on estrogen-induced HMG in rats. RuXian-I had no effect on the body weight and food intake of HMG rats and had no toxic effects on the five organs (heart, lung, spleen, and kidney). RuXian-I reduced the diameter and height of nipple, organ index, and pathological changes and alleviated the sex hormone levels oh HMG; RuXian-I reduced the upregulation of TCTP, Mcl-1, and Bcl-xL in breast tissue of mammary gland hyperplasia and increased the downregulation of p53, Bax, caspase-9, and caspase-3 protein. RuXian-I has an effective therapeutic activity on HMG rats, and its possible therapeutic mechanism is closely related to antiapoptosis protein TCTP-regulated apoptosis.

## 1. Introduction

Hyperplasia of mammary gland (HMG) is one of the benign breast diseases, also known as breast dysplasia, which is classified as “Ru Pi” by Chinese medicine [1], accounting for more than 70% of all breast diseases [2]. In modern medicine breast hyperplasia is the most common middle-aged women's disease [3]. Due to the fast pace of modern life, the incidence of HMG has increased year by year with the increase of work pressure and competitive pressure [4]. Women with HMG have a significantly increased risk of breast cancer [5]. In some parts of China, the incidence of HMG is approaching 30% [6], which poses a serious threat to women's physical and mental health and quality of life.

At present, clinical trials on the treatment of HMG are neither exact nor very effective. Hormone therapy or endocrine therapy is often used to relieve symptoms in a short period of time [7]. However, the side effects and complications of these therapies are severe and the efficacy of long-term use is questionable. Most people are difficult to accept surgery and also prone to recurrent symptoms [8]. Therefore, many patients need complementary or alternative therapies, such as Mongolian medicine [9]. Modern clinical research shows that traditional Mongolian medicines, through the whole regulation, multilink, multiway, and multitarget to work together, have reliable function and lower toxicity in the treatment of HMG. However, the traditional Mongolian medicine RuXian-I is an empirical formula specifically used for the treatment of HMG in clinic based on the principles of traditional Mongolian medicine. RuXian-I has been used clinically for many years and can effectively alleviate the symptoms of HMG.

Through two-dimensional difference gel electrophoresis (2D-DIGE), the expression changes of 17 proteins have been identified in the RuXian-I treatment of HMG by using the method of proteomics. Among them, RuXian-I significantly reduced the expression of translationally controlled tumor protein (TCTP) in HMG rats [10]. TCTP was found to be a highly conserved protein encoded by the* TPT1* gene in all eukaryotes [11]. Originally found in Ehrlich ascites tumor cells, no homology with other proteins was found [12]. Because of its versatility, it is also known as histamine release factor,* TPT1, p23*, or* fortilin* [13].

TCTP is expressed in the nucleus and cytoplasm of cells and is regulated by extracellular stimuli [13]. It is expressed both intracellularly and extracellularly and involves many biological processes such as development [14], cell cycle [12], cell growth [15], protein synthesis [16], cytoskeleton [17], immune response [18], cell death [19], and induction of pluripotent stem cells [20]. In addition, proteins interact with themselves or with other proteins. TCTP interacts with other members of myeloid cell leukemia 1 (Mcl-1) [21] and B-cell lymphoma 2 (Bcl-2). Apoptosis plays an important role. It destabilizes the tumor suppressor p53, antagonizes Bax, activates Bcl-xL, and prevents caspase-3-dependent apoptosis [22].

TCTP is involved in the regulation of basic cellular processes and is characterized by high level of expression in several tumor types [15]. And its high level of expression is also associated with poorly differentiated invasive breast cancer [23]. However, our previous studies indicated that TCTP is highly expressed in HMG. This high expression may be reduced after treatment with Mongolian medicine, which is treated with RuXian-I [10]. The purpose of this study is to reveal the TCTP protein and its possible mechanism in the treatment of HMG with RuXian-I.

## 2. Materials and Methods

### 2.1. Animal Experiment

The traditional Mongolian medicine RuXian-I, provided by the affiliated hospital of Inner Mongolia University for Nationalities, consists of 30 herbs [10]. All medicinal materials were purchased by Anhui Zhongmeitang Chinese Herbal Pieces Co., Ltd (NM2016-12). All voucher specimens are deposited in a herbarium center of the affiliated hospital of Inner Mongolia University for Nationalities (IMUN-20160912). Firstly, the crude herbs were crushed into small pieces. Then these small pieces were processed by one of following two methods: (1) extracting with distilled water and then filtering, concentrating and evaporating the extract, leaving a dry powder; (2) crushing by ball milling and sifting to obtain a fine powder. The two kinds of powder were properly mixed and pills prepared by rolling a soft mass into a round shape. After drying, coating, polishing, sterilization and packaging, the product was considered complete. The drug was identified as a qualified product by Professor Guihua Bao from the Mongolian Medical College of Inner Mongolia University for Nationalities. The product powder was dissolved in distilled water to make three doses required for the experiment. The drug was stored in a refrigerator at 4°C and taken out to room temperature before use. The samples were shaken well before use.

In accordance with the Guide for the Animal Care and Use of Laboratory Animals published by the National Institutes of Health (NIH Publications No. 85–23, revised 1996), all experimental protocols and animal handling procedures were approved by the Animal Care and Use Committee of Inner Mongolia University for the Nationalities (Ethics committee number: 0010/2016). Fifty nonpregnant female Wistar rats weighing 200 ± 20 g were supplied by the experimental animal center of Jilin University (SCXK-2016-05). The rats were raised in plastic cages with temperature- and humidity-controlled room (20~25°C and 50%~60%, respectively) under a 12 h light/dark cycle (the illumination time: 6:00 ~18:00), with rodent chow and water ad libitum, acclimation for a week.

Rats were randomly divided into two groups according to their body weight: 10 rats in the control group and 40 rats in HMG group. Rats in control group were intramuscularly injected with olive oil (Sigma-Aldrich) and rats in HMG group were intramuscularly injected with 0.5mg•kg^−1^•d^−1^ estradiol benzoate (Hangzhou Animal Medicine Factory) for consecutive 25 days, followed by intramuscular injection of 4 mg•kg^−1^•d^−1^ of progesterone (Zhejiang Xianju Pharmaceutical Co., Ltd) for 5 days. Then, according to the rat's nipple diameter, the rats with HMG were randomly divided into 4 groups after successful replication of the model. After grouping five groups of rats, the specific experimental administration is as follows. Normal saline (0.1mL•kg^−1^•d^−1^) was administrated with normal saline for 30 days in control group and model group (10 rats, respectively). And drug (0.5, 1.0, and 3.0g•kg^−1^•d^−1^, 10 rats, respectively) was administrated for 30 days continuously in RuXian-I low, medium, and high-dose group (respectively).

All experimental animals during the experiment had normal drinking water diet. The daily living condition of each group was observed and the food intake weight was weighed. The body weight of rats and the nipples diameter and height of the second pair in rats were measured.

After continuous intragastric administration, the animals were fasted for 12 hours and were anesthetized with pentobarbital (140 mg•kg^−1^) intraperitoneally. After sample collection, the rats were euthanized by cervical dislocation. Place the rat on the lid of the feeding box, then study to grasp the tail of the mouse with your right hand, pull back the tail slightly, quickly press the thumb and the index finger of the left hand on the head, and dislocate the neck of the mouse with both hands.

### 2.2. Organ Index

Rat body weight after anesthesia, rat organs uterus, ovary, thymus, and spleen were collected and weighed and calculated: organ index = organ weight/body weight.

### 2.3. Histopathology

Breast and heart, lung, spleen, and kidney tissue in all groups were fixed in 4% paraformaldehyde for 24 hours. The fixed tissue was rinsed with distilled water and then dehydrated in 70%, 80%, 90%, 95%, and 100%. The dehydrated tissue was placed in a mixture of absolute ethanol and xylene twice, each time for 30 min, then immersed in melted paraffin, and placed in an oven at 60°C for 1 h. The baptist tissue block and paraffin were poured into the embedding frame, and the block was frozen and frozen. The block was sliced with a microtome and sliced to a thickness of 4 *μ*m. Use eye tweezers to tweezer slices gently tiling the pieces in water at 40-45°C, flatten the wrinkled wax strips naturally, and place the slices in an oven for drying: xylene soak 2 times each time 5 min, ethanol immersion 2 times each 5 min, 95%, 90%, 80%, and 70% ethanol in turns every 3 min, and distilled water rinse. Followed by hematoxylin staining 5 min, there are tap water rinse 3 min, eosin staining after dehydration 2 min, 95% ethanol 40s, 100% ethanol 2 times each 5 min, and xylene 2 times each 5 min. Overnight at 60°C, dried with a neutral gum seal. Microscopic observation of slices and pictures were taken.

### 2.4. Immunohistochemistry

In Elivision two-step method (Fujian Mai new Biological Technology Co., Ltd.), the specific steps are as follows: paraffin slices were placed in an oven at 68°C 2 h, with dewaxing to water, and washed with PBS buffer 3 times, each 3 min. The citrate buffer (pH6.0) was heated to boiling, the dewaxed and hydrated tissue sections were placed on a high-temperature plastic slicing rack, placed in a boiling buffer, and boiled 10 min, natural Ling. However, remove the slide from the buffer, rinse twice with distilled water, and rinsed 3 times with PBS buffer for 3 minutes each. Elimination of endogenous peroxidase: 3% H_2_O_2_ is incubated at room temperature for 10 min and washed 3 times with PBS buffer, each 3 min. Remove the PBS buffer, add appropriate diluted TCTP antibody (Cell Signal Technology, Inc), incubate for 2 h at room temperature, and wash 3 times with PBS buffer, each 3 min. Remove the PBS buffer, add polymer enhancer (avidin), incubate at room temperature for 20 min, and wash with PBS buffer 3 times for 3 min each time. Remove the PBS buffer, dropping enzyme-labeled anti-mouse/rabbit polymer (biotin-horseradish peroxidase), and incubate at room temperature for 30 min and PBS buffer is rinsed 3 times, each 3 min. Remove the PBS buffer and drip freshly prepared DAB chromogenic solution, under a microscope for 5 min. Counterstaining: stained with hematoxylin, 0.1% HCl is rinsed with tap water, blued, sliced, and dehydrated by gradient alcohol: Transparent and Enclosed: xylene transparent, Neutral Glue Enclosed. Microscopically observe the slices and photograph the pictures, and perform semiquantitative analysis with Image-Pro Plus 6 image analysis software.

### 2.5. Western Blotting

The mammary gland tissues were rapidly separated on ice plate, frozen in liquid nitrogen, and stored at −80°C. Total protein was extracted from tissues of the mammary gland randomly selected from each group and their content was detected by BCA protein assay kit (Beijing Tiangen Biotech Co., Ltd). Total protein (20 *μ*g) per sample was resolved by 12% sodium dodecyl sulfate polyacrylamide gel electrophoresis (SDS-PAGE) and transferred to PVDF membrane (Millipore). PVDF membranes were incubated in 5% nonfat dry milk for 2 h. TCTP (1:1000), Mcl-1 (1:1000, Cell Signal Technology, Inc), caspase-3 (1:1000, Cell Signal Technology, Inc), caspase-9 (1:1000, Cell Signal Technology, Inc), Bax (1:1000, Abcam), Bcl-xL (1:200, Abcam), and p53 (1:1000, OriGene Technologies, Inc) antibodies were added and then the corresponding Peroxidase-conjugated affinipure goat anti-rabbit IgG (1:5000, Beijing fir ZhongshanGoldenbridge Biotechnology Corporation) and rabbit anti-mouse IgG (1:5000, Beijing fir ZhongshanGoldenbridge Biotechnology Corporation) secondary antiserum were detected. ECL (Millipore Corporation) was used to detect imprinted membrane (Millipore Corporation). The blots were identified for band densities using Image J 1.45s software.

### 2.6. Radioimmunoassay

The level of serum estradiol (E_2_), progesterone (P), and testosterone (T) was measured in accordance with the instructions of the corresponding RIA kit.

### 2.7. Statistical Analysis

All values were expressed as the means ± SD. Data was analyzed using the SPSS17.0 statistical software. Data among the groups were analyzed by homogeneity test of variances expressed in forms of standard deviation. The comparison of multiple sample means was used with One-Way ANOVA test. All experiments were performed at least three times. p<0.05 was accepted as significant. The statistic and graphic software GraphPad Prism (Version 5, GraphPad Software, Inc., La Jolla, CA) was used for all statistical and graphic analysis.

## 3. Results

### 3.1. Changes of the Nipple Height and Diameter

**Figure 1:**
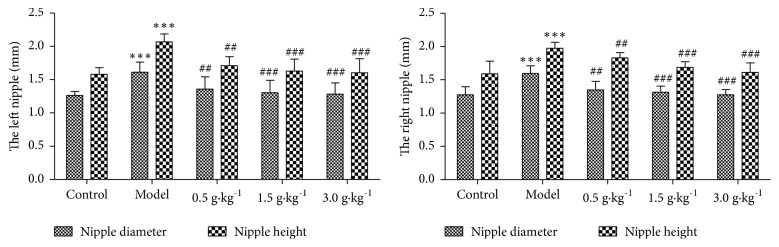
Changes of left and right second pair of nipple diameter and height in each group (^*∗∗∗*^p <0.001 vs. Control, ^##^p <0.01 or ^###^p <0.001 vs. Model; n = 10).

At the end of the experiment, second pair of nipple diameter and height in the rat was detected. Compared with the control group, nipple diameter and height significantly increased (P <0.001). However, after the RuXian-I treatment, the results showed that nipple diameter and height showed a dose-dependent alleviation compared with the model group (p <0.01, Figure 1).

### 3.2. Changes of Organ Index

**Figure 2:**
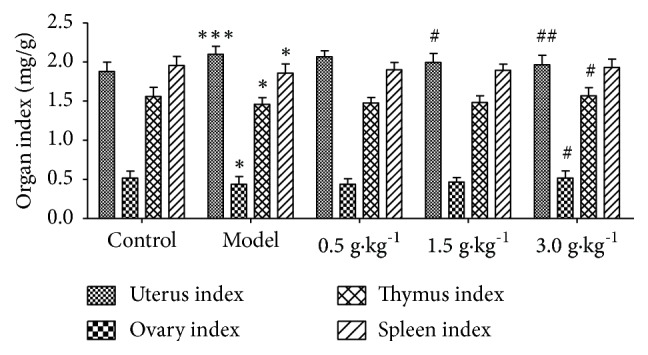
Changes of the uterus, ovary, thymus, and spleen index in each group rats (^*∗*^P <0.05 or ^*∗∗∗*^p <0.001 vs. Control, ^#^p <0.05 or ^##^p <0.001 vs. Model; n = 10).

In this study, we evaluated the change of organ index in rats. The results showed that compared with control group, the uterus index increased significantly in model group (p <0.001) and decreased in ovary, thymus, and spleen index (p <0.05). However, after treatment with Mongolian RuXian-I at medium, high, and low dose, the index of the uterus decreased in a dose-dependent manner. Ovarian, thymus, and spleen index increased, but this is not obvious (Figure 2).

### 3.3. Changes of Serum Sex Hormone Levels

**Figure 3:**
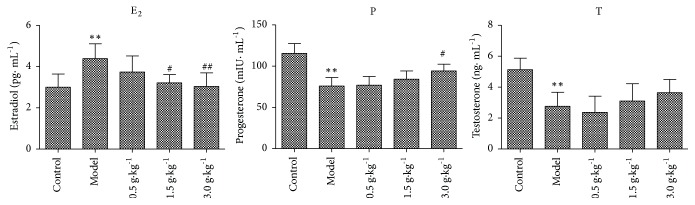
Changes of serum estrogen E_2_, P, and T levels in each group rats. (^*∗∗*^p <0.01 vs. Control, ^#^p <0.05 or ^##^p <0.001 vs. Model; n = 3).

Serum 3 sex hormone levels were assessed by radioimmunoassay. The results showed that compared with control group, the level of serum E_2_ in HMG group was significantly increased (p <0.01) and the levels of P and T were significantly decreased (p <0.01). However, after treatment with RuXian-I at low, medium, and high dose, the level of E_2_ showed a dose-dependent decrease; P and T showed a small and insignificant dose-dependent increase (Figure 3).

### 3.4. Changes of Breast Histopathology

The pathological changes of mammary gland tissue in each group were evaluated by HE staining. Microscopically, we can see that the control group had normal histological structure of mammary gland duct and acinus. The breast lobular was small in size and had fewer lobes. The acinar was arranged closely around the mammary duct without any expansion. The catheter was single or double Cubic epithelial cells, wall is thin, and duct and acinar are rich in adipose tissue and connective tissue, including small blood vessels.

HMG group showed typical mammary gland hyperplasia, manifested as breast lobular volume was significantly larger, more leaves, the number and size of the catheter and acinar significantly increased, acinar dilatation significantly, a large number of catheter cavity loss epithelial cells and secretions, the number of duct wall cells increased significantly thicker, accompanied by a large number of irregularly arranged epithelial cells, connective tissue and adipose tissue decreased.

**Figure 4:**
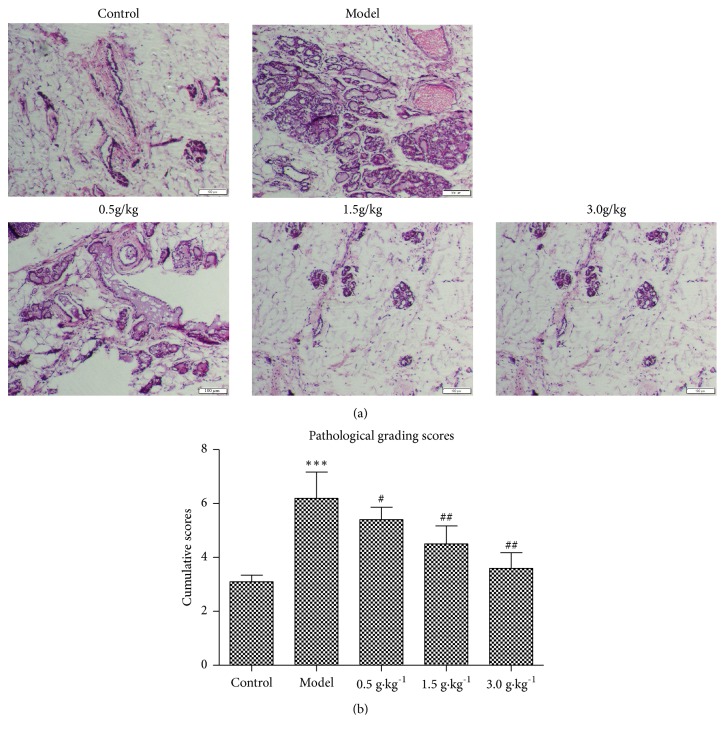
HE staining (a) and cumulative pathological grading (b) in breast tissues of each group (magnification 400×, ^*∗∗∗*^p <0.001 vs. Control, ^#^p <0.05 or ^##^p <0.01 vs. Model; n = 3).

Compared with the model group, the numbers and sizes of mammary lobular and alveolar of RuXian-I treatment group were reduced, the expansion of the acinar cavity was reduced, the volume of the catheter and the lumen became smaller, and the secretion in the cavity also decreased. Especially in the high-dose group (3.0 g•kg^−1^), the number of leaflets and acinar significantly decreased, the acinar cavity dilated slightly, and the catheter lumen was less or no secretions were found. These results indicate that RuXian-I showed a dose-dependent manner in improving the histopathological changes of breast tissue in HMG rats. In short, from the histopathological observation, it can be seen that the RuXian-I has a high therapeutic effect.  Figure 4(a) shows histone HE staining changes, and Figure 4(b) shows cumulative histological grading of breast tissue.

### 3.5. Changes of TCTP Protein Levels

In order to detect TCTP protein levels in breast tissues of rats in each group, immunohistochemistry and Western blotting methods were used to evaluate the TCTP protein levels.

**Figure 5:**
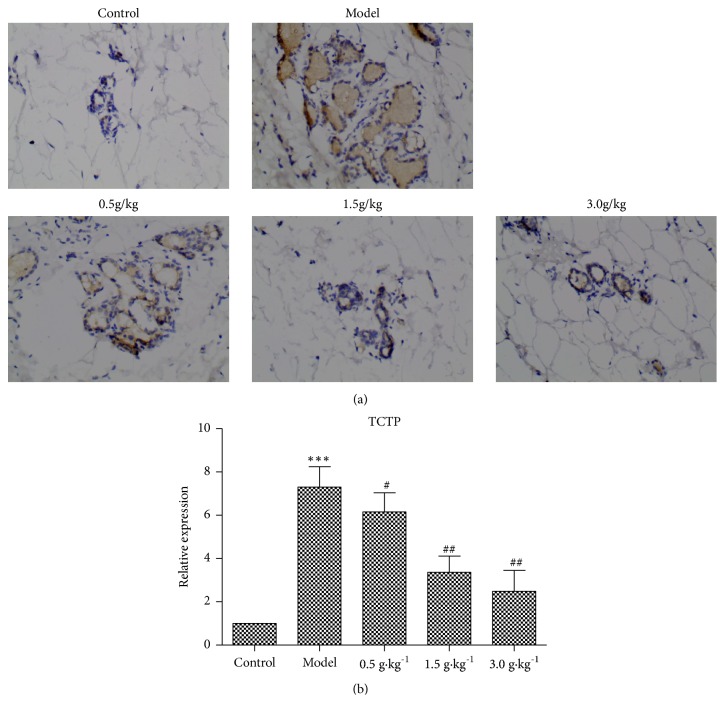
Immunohistological staining (a) and relative expression quantification (b) of TCTP protein in breast tissues of each group (magnification 400×, ^*∗∗∗*^p <0.001 vs. Control, ^#^p <0.05 or ^##^p <0.01 vs. Model; n = 3).

Microscopically, immunohistochemistry results showed that the expression of TCTP protein in breast tissue of control group was weakly positive, which was expressed in brown or brown granular. Compared with control group, the expression of TCTP protein in HMG group was significantly increased, with yellow or brown particles having larger staining area and deep staining; RuXian-I TCTP-positive cells in each treatment group weakened expression, especially in the high-dose group, significantly decreased.  Figure 5(a) shows the immunohistochemical staining of TCTP protein in breast tissue, and Figure 5(b) shows the relative changes of TCTP protein expression.

**Figure 6:**
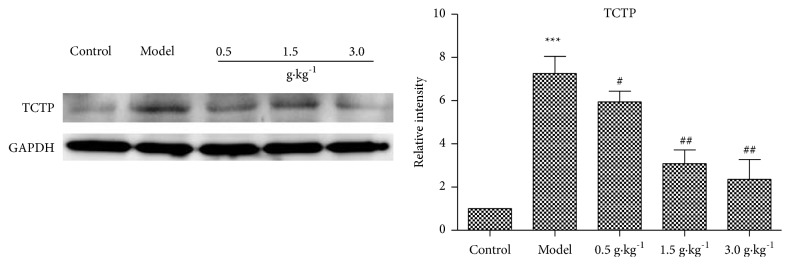
Western blotting results of TCTP protein in breast tissues of each group (^*∗∗∗*^p <0.001 vs. Control, ^#^p <0.05 or ^##^p <0.01 vs. Model; n = 3).

Western blotting showed that the expression of TCTP protein in mammary glands of HMG group was significantly higher than that of control group (p <0.01), while RuXian-I treatment group decreased the expression of TCTP protein, especially the high-dose group (3.0 g • kg^−1^) (p <0.01; Figure 6).

### 3.6. Changes of Apoptosis-Related Protein Levels

**Figure 7:**
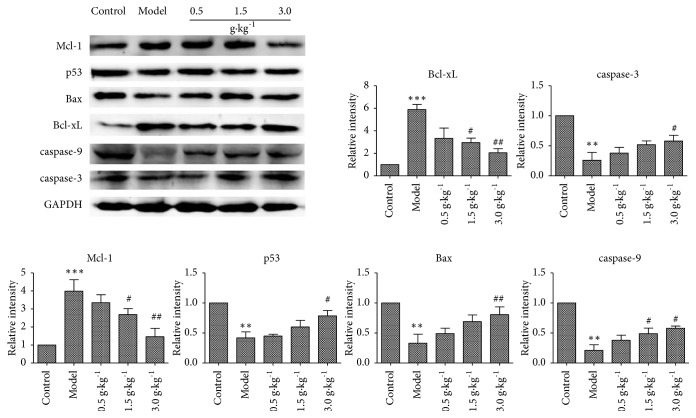
Western blotting results of apoptosis-related proteins Mcl-1, p53, Bax, Bcl-xL, caspase-9, and caspase-3 in breast tissues of each group (^*∗∗*^p <0.01 or ^*∗∗∗*^p <0.001 vs. Control, ^#^p <0.05 or ^##^p <0.01 vs. Model; n = 3).

In order to explore the possible molecular mechanism of RuXian-I treatment of HMG model, the expression of apoptosis-related protein was detected by Western blotting. After inducing by estrogen and progesterone, the expressions of proapoptotic proteins caspase-3, caspase-9, and Bax and tumor suppressor protein p53 were significantly downregulated (p <0.01), whereas Mcl-1 and Bcl-xL were significantly upregulated (P <0.001). However, the expression levels of caspase-3, caspase-9, and Bax and the tumor regulatory protein p53 in HMG rats treated with RuXian-I increased in a dose-dependent manner, and the levels of Mcl-1 and Bcl-xL were significant in a dose-dependent manner (Figure 7).

## 4. Discussion

Based on the previous studies, RuXian-I was evaluated as a hormonal regulator, which has protective and therapeutic effects on HMG rats by regulating the function of the endocrine system and the immune system. The study showed rat body weight, food intake and five organs of heart, liver, spleen, lung, and kidney pathological examination in RuXian-I treatment of HMG rats are not found to have any change, indicating safety of RuXian-I.

Estrogen and progestin are key hormones for ductal extension and branching in adolescent mammary ducts [24]. Hormone levels can be a type of reactivating disease and an effective treatment option [25]. Sex hormones have an important impact on the immune system, regulating the growth and development of lymphoid tissues. The thymus is the major target organ of the immune system's central hormone. Spleen is a place where lymphocytes reside and proliferate and provide specific cellular and humoral immunity [26]. RuXian-I, as hormonal regulator of mammary gland hyperplasia rats, decrease the concentration of E_2_ and increase the concentration of P and T; RuXian-I have different degrees in reducing the height and diameter of the nipple and a good recovery of breast lobular volume, breast lobular, and breast lobular number; RuXian-I can also increase the thymus or spleen or ovarian index of HMG rats, decrease the uterine index, and alleviate the proliferation of breast tissue. So RuXian-I have protective effect on HMG rat thymus, spleen, uterus, ovary, and breast. These results indicate that RuXian-I exerts anti-HMG by regulating the immune system and hormone levels.

Previous studies identified increasing of TCTP expression in HMG rats. TCTP is found to be involved in tumor regression such as prostate cancer, lung cancer, leukemia, erythroleukemia, glioma, lymphoma, squamous cell carcinoma, colon cancer, liver cancer, laryngeal cancer, and melanoma [15, 27]. In a wide range of cancers, higher levels of TCTP expression are found in tumors compared with the corresponding normal tissues [27]. TCTP expression has been observed to be 2.7-fold decreased during cell differentiation of colon cancer Caco2 cells [28], indicating the importance of the TCTP protein for differentiation. TCTP protein is also closely related to the occurrence and development of breast cancer [29]. Downregulation of TCTP in breast cancer MCF-7 and T47D cells leads to cell recombination. In addition, proteins are significantly downregulated during tumor reversion and are associated with aggressive clinical and pathologic parameters of breast cancer. This suggests that TCTP may serve as a prognostic factor for breast cancer [15]. TCTP is involved in cell differentiation and tumor reversal as a potential target for anticancer therapies [15]. The results of this study show that RuXian-I dose-dependently reduced the expression of TCTP protein in HMG rats, and the results were also based on previous studies [10]. Therefore, TCTP is highly expressed in HMG rats and its increased expression may be the key protein that leads to HMG.

**Figure 8:**
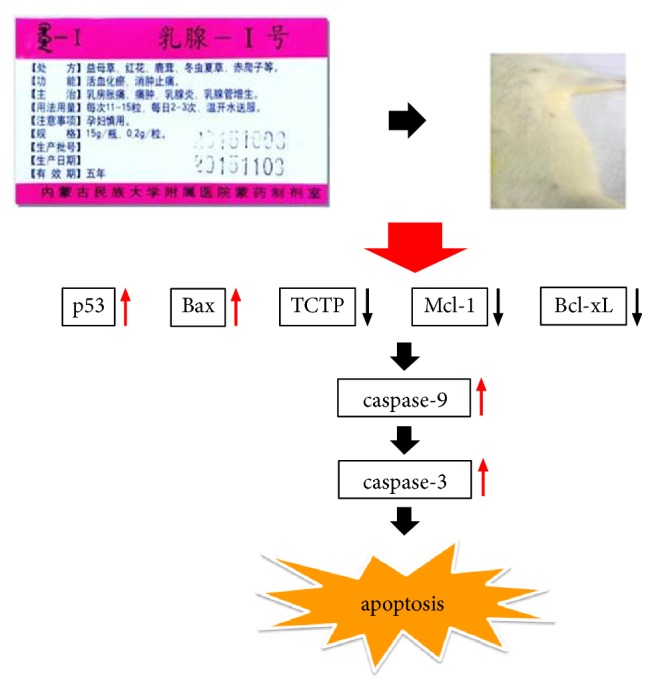
RuXian-I on the proliferation of HMG cells apoptosis-induced signaling pathway.

TCTP plays a key role in the regulation of apoptosis. TCTP inhibits Mcl-1 degradation by blocking its ubiquitination to regulate antiapoptotic activity [23] and interacts with other antiapoptotic proteins such as Bcl-xL in the Bcl-2 family. However, TCTP and Mcl-1 also independently protect cells from apoptosis [30]. TCTP interacts with other antiapoptotic proteins from the Bcl-2 family such as Bcl-xL and Bax [22]. The N-terminal region of TCTP interacts with the Bcl-2 homology domain 3 site of Bcl-xL to mediate inhibition of apoptosis [22]. It has also been found that Arg21 in the N-terminal region of TCTP is critical for the binding of TCTP to Mcl-1 [31]. Homodimerization of proapoptotic Bax is essential for its apoptotic activity. TCTP blocks Bax apoptosis by inserting mitochondrial membrane and inhibiting Bax dimerization. Unlike Mcl-1 and Bcl-xL, TCTP does not bind Bax directly [32]. It is well known that the tumor suppressor p53 protein is a transcription factor that regulates the transcription of many genes. It is well known that the tumor suppressor p53 protein is a transcription factor that regulates the transcription of many genes. It activates the transcription of DNA repair genes in DNA damage by regulating genes involved in cell cycle and apoptosis, such as Bax and Bcl-2 [33]. p53 promotes apoptosis, whereas TCTP prevents apoptosis by inhibiting p53 transcription. TCTP binds to p53 and prevents caspase-3-dependent apoptosis by destabilizing the protein. Active caspase-3 acts as a regulator of TCTP and p53 controls the production of exosomes and the release of TCTP and plays a key role in the release of nanovesicles from TCTP in apoptosis. The present results show that the expressions of TCTP, Mcl-1, and Bcl-xL in HMG rats are increased while the levels of p53, Bax, caspase-9, and caspase-3 are decreased. HMG is closely related to apoptosis. When cysteine enzyme is activated, resistance to apoptosis leads to the occurrence and development of mammary gland hyperplasia and even activates oncogenes leading to mammary tumor formation. However, RuXian-I could decrease the protein upexpression of TCTP, Mcl-1, and Bcl-xL in HMG rats in a dose-dependent manner and increase the downexpression levels of p53, Bax, caspase-9, and caspase-3 in a dose-dependent manner. Thus, one of the molecular mechanisms involved in the inhibition of breast cell proliferation in HMG may be through decreasing the protein expression of Mcl-1, Bcl-xL, and TCTP, while another mechanism may be through increasing p53, Bax, caspase-9, and caspase-3, thereby inducing apoptosis (Figure 8). However, the exact mechanism by which TCTP protein affects apoptosis of breast hyperplasia needs further elucidation.

In short, molecular mechanism of RuXian-I treatment of HMG is complex; in our previous research work and our hospital clinical breast hyperplasia patients medication, it has been proved that RuXian-I can be effective in treating HMG and found no obvious side effects. This study also demonstrated that TCTP protein is closely related to RuXian-I treatment of HMG, possibly as a target protein for its treatment, and may be involved in the molecular mechanism of TCTP-mediated apoptosis. This study further elucidated the molecular mechanism of RuXian-I treatment of HMG.

## Data Availability

All data generated or analyzed during this study are included in this article. However, further details are available from the corresponding author on reasonable request.

## References

[1] Zheng J., Zhao Y., Wang Y. (2013). The infrared radiation temperature characteristic of acupoints of mammary gland hyperplasia patients. *Evidence-Based Complementary and Alternative Medicine*.

[2] Zhou L. N. (2010). Chinese medicine treatment of breast hyperplasia. *Zhong Yi Yao Dao Bao*.

[3] Chen T., Li J., Chen J., Song H., Yang C. (2015). Anti-hyperplasia effects of Rosa rugosa polyphenols in rats with hyperplasia of mammary gland. *Environmental Toxicology and Pharmacology*.

[4] Castells X., Domingo L., Corominas J. M. (2015). Breast cancer risk after diagnosis by screening mammography of nonproliferative or proliferative benign breast disease: a study from a population-based screening program. *Breast Cancer Research and Treatment*.

[5] Hayes M. M., Konstantinova A. M., Kacerovska D. (2016). Bilateral gigantomastia, multiple synchronous nodular pseudoangiomatous stromal hyperplasia involving breast and bilateral axillary accessory breast tissue, and perianal mammary-type hamartoma of anogenital mammary-like glands: a case report. *American Journal of Dermatopathology*.

[6] Mo D. H. (2011). The status of breast hyperplasia and its related factors among women of childbearing age in urban and suburban. *Modern Medicine Journal of China*.

[7] Jordan V. C. (2015). The new biology of estrogen-induced apoptosis applied to treat and prevent breast cancer. *Endocrine-Related Cancer*.

[8] Nguyen C. V., Albarracin C. T., Whitman G. J., Lopez A., Sneige N. (2011). Atypical ductal hyperplasia in directional vacuum-assisted biopsy of breast microcalcifications: Considerations for surgical excision. *Annals of Surgical Oncology*.

[9] Oyunchimeg B., Hwang J. H., Ahmed M., Choi S., Han D. (2017). Complementary and alternative medicine use among patients with cancer in Mongolia: a National hospital survey. *BMC Complementary and Alternative Medicine*.

[10] Wang Z.-C., E D., Batu D.-L., Saixi Y.-L., Zhang B., Ren L.-Q. (2011). 2D-DIGE proteomic analysis of changes in estrogen/progesterone-induced rat breast hyperplasia upon treatment with the mongolian remedy RuXian-I. *Molecules*.

[11] Oh Y. T., Ahn C.-S., Jeong Y. J. (2013). Aspergillus nidulans translationally controlled tumor protein has a role in the balance between asexual and sexual differentiation and normal hyphal branching. *FEMS Microbiology Letters*.

[12] Brioudes F., Thierry A.-M., Chambrier P., Mollereau B., Bendahmane M. (2010). Translationally controlled tumor protein is a conserved mitotic growth integrator in animals and plants. *Proceedings of the National Acadamy of Sciences of the United States of America*.

[13] Nagano-Ito M., Ichikawa S. (2012). Biological effects of mammalian translationally controlled tumor protein (TCTP) on cell death, proliferation, and tumorigenesis. *Biochemistry Research International*.

[14] Santana J., Marzolo M.-P. (2017). The functions of Reelin in membrane trafficking and cytoskeletal dynamics: Implications for neuronal migration, polarization and differentiation. *Biochemical Journal*.

[15] Seo E.-J., Fischer N., Efferth T. (2017). Role of TCTP for cellular differentiation and cancer therapy. *Results and Problems in Cell Differentiation*.

[16] Bonhoure A., Vallentin A., Martin M. (2017). Acetylation of translationally controlled tumor protein promotes its degradation through chaperone-mediated autophagy. *European Journal of Cell Biology*.

[17] Tsarova K., Yarmola E. G., Bubb M. R. (2010). Identification of a cofilin-like actin-binding site on translationally controlled tumor protein (TCTP). *FEBS Letters*.

[18] Wu W., Wu B., Ye T. (2013). TCTP is a critical factor in shrimp immune response to virus infection. *PLoS ONE*.

[19] Hoepflinger M. C., Reitsamer J., Geretschlaeger A. M., Mehlmer N., Tenhaken R. (2013). The effect of translationally controlled tumour protein (TCTP) on programmed cell death in plants. *BMC Plant Biology*.

[20] Acunzo J., Baylot V., So A., Rocchi P. (2014). TCTP as therapeutic target in cancers. *Cancer Treatment Reviews*.

[21] Su C.-C. (2014). Tanshinone IIA inhibits human gastric carcinoma AGS cell growth by decreasing BiP, TCTP, Mcl-1 and Bcl-xL and increasing Bax and CHOP protein expression. *International Journal of Molecular Medicine*.

[22] Thébault S., Agez M., Chi X. (2016). TCTP contains a BH3-like domain, which instead of inhibiting, activates Bcl-xL. *Scientific Reports*.

[23] Seo E.-J., Efferth T. (2016). Interaction of antihistaminic drugs with human translationally controlled tumor protein (TCTP) as novel approach for differentiation therapy. *Oncotarget *.

[24] Miao X., Chen Y.-B., Xu S.-L. (2013). TCTP overexpression is associated with the development and progression of glioma. *Tumor Biology*.

[25] Stierum R., Gaspari M., Dommels Y. (2003). Proteome analysis reveals novel proteins associated with proliferation and differentiation of the colorectal cancer cell line Caco-2. *Biochimica et Biophysica Acta (BBA) - Proteins and Proteomics*.

[26] Chien S.-Y., Kuo S.-J., Chen D.-R., Su C.-C. (2013). Sann-Joong-Kuey-Jian-Tang decreases the protein expression of Mcl-1 and TCTP and increases that of TNF-*α* and Bax in BxPC-3 pancreatic carcinoma cells. *International Journal of Molecular Medicine*.

[27] Zhang D., Li F., Weidner D., Mnjoyan Z. H., Fujise K. (2002). Physical and functional interaction between myeloid cell leukemia 1 protein (MCL1) and fortilin. *The Journal of Biological Chemistry*.

[28] Feng H., Zhu Z., Cao W. (2018). Foot-and-mouth disease virus induces lysosomal degradation of NME1 to impair p53-regulated interferon-inducible antiviral genes expression. *Cell Death & Disease*.

[29] Villegas J. A., Gradolatto A., Truffault F. (2018). Cultured human thymic-derived cells display medullary thymic epithelial cell phenotype and functionality. *Frontiers in Immunology*.

[30] Sirois I., Raymond M., Brassard N. (2011). Caspase-3-dependent export of TCTP: a novel pathway for antiapoptotic intercellular communication. *Cell Death & Differentiation*.

[31] Lespagnol A., Duflaut D., Beekman C. (2008). Exosome secretion, including the DNA damage-induced p53-dependent secretory pathway, is severely compromised in TSAP6/Steap3-null mice. *Cell Death & Differentiation*.

[32] Arendt L. M., Kuperwasser C. (2015). Form and function: how estrogen and progesterone regulate the mammary epithelial hierarchy. *Journal of Mammary Gland Biology and Neoplasia*.

[33] Li X., Xin P., Wang C., Wang Z., Wang Q., Kuang H. (2017). Mechanisms of traditional chinese medicine in the treatment of mammary gland hyperplasia. *American Journal of Chinese Medicine*.

